# Computational investigations on target-site searching and recognition mechanisms by thymine DNA glycosylase during DNA repair process: Structural dynamics of TDG at atomic resolution

**DOI:** 10.3724/abbs.2022050

**Published:** 2022-05-18

**Authors:** Lingyan Wang, Kaiyuan Song, Jin Yu, Lin-Tai Da

**Affiliations:** 1 Key Laboratory of Systems Biomedicine (Ministry of Education) Shanghai Center for Systems Biomedicine Shanghai Jiao Tong University Shanghai 200240 China; 2 Department of Physics and Astronomy Department of Chemistry NSF-Simons Center for Multiscale Cell Fate Research University of California Irvine CA 92697 USA

**Keywords:** DNA repair, DNA glycosylase, Markov state model, molecular dynamics simulation

## Abstract

DNA glycosylase, as one member of DNA repair machineries, plays an essential role in correcting mismatched/damaged DNA nucleotides by cleaving the N-glycosidic bond between the sugar and target nucleobase through the base excision repair (BER) pathways. Efficient corrections of these DNA lesions are critical for maintaining genome integrity and preventing premature aging and cancers. The target-site searching/recognition mechanisms and the subsequent conformational dynamics of DNA glycosylase, however, remain challenging to be characterized using experimental techniques. In this review, we summarize our recent studies of sequential structural changes of thymine DNA glycosylase (TDG) during the DNA repair process, achieved mostly by molecular dynamics (MD) simulations. Computational simulations allow us to reveal atomic-level structural dynamics of TDG as it approaches the target-site, and pinpoint the key structural elements responsible for regulating the translocation of TDG along DNA. Subsequently, upon locating the lesions, TDG adopts a base-flipping mechanism to extrude the mispaired nucleobase into the enzyme active-site. The constructed kinetic network model elucidates six metastable states during the base-extrusion process and suggests an active role of TDG in flipping the intrahelical nucleobase. Finally, the molecular mechanism of product release dynamics after catalysis is also summarized. Taken together, we highlight to what extent the computational simulations advance our knowledge and understanding of the molecular mechanism underlying the conformational dynamics of TDG, as well as the limitations of current theoretical work.

## Introduction

Human genome is constantly under threat from external or internal damaging agents, such as the ultraviolet (UV) radiation and/or detrimental factors from host cells that can lead to chemical modifications of the nucleobases, including deamination, oxidation and alkylation [1]. These DNA lesions/errors can cause devastating diseases via altering the genome stability, including cancer and premature aging [2]. Fortunately, the cells have evolved advanced strategies to correct the above damages via various repairing mechanisms according to the nature of the lesions. Among which, base excision repair (BER), as one of the critical DNA repair pathways, targets and then corrects the damaged or mismatched nucleobases via consecutively recruiting and employing several enzymes, including DNA glycosylase, endonuclease, DNA polymerase and DNA ligase, along with other accessory proteins [3].

To date, at least 11 DNA glycosylases have been found in human cells, with each capable of interrogating varied (or overlapped) chemical groups [4]. As one critical member, thymine DNA glycosylase (TDG) is responsible for cleaving the N-glycosidic bond of several mismatched or chemically damaged nucleotides by initiating the BER pathway, thereby generating an apurinic/apyrimidinic (AP) site [ 5, 6] . For example, TDG can specifically target to the G:T or U:G mispairs as well as several modified uracil derivatives, including 5-hydroxymethyl uracil (5hmU), 5-formyluracil (FoU), and 5-halogenated uracil ( *e*. *g*., 5FU, 5ClU, 5BrU, and 5IU) [ 1, 7– 9] . More importantly, TDG has also been found to participate in regulating epigenetic patterns by involving an active DNA demethylation process. Notably, TDG can readily excise the modified forms of 5-methylcytosine (5mC), such as 5-formylcytosine (5fC) and 5-carboxycytosine (5caC), instead of 5mC [ 10, 11] . Therefore, TDG serves as a critical factor for maintaining human genome integrity and regulating epigenetic marks, also being considered as a potential drug target, *e*. *g*., for melanoma and breast cancer [ 12– 14] .

**Figure 1:**
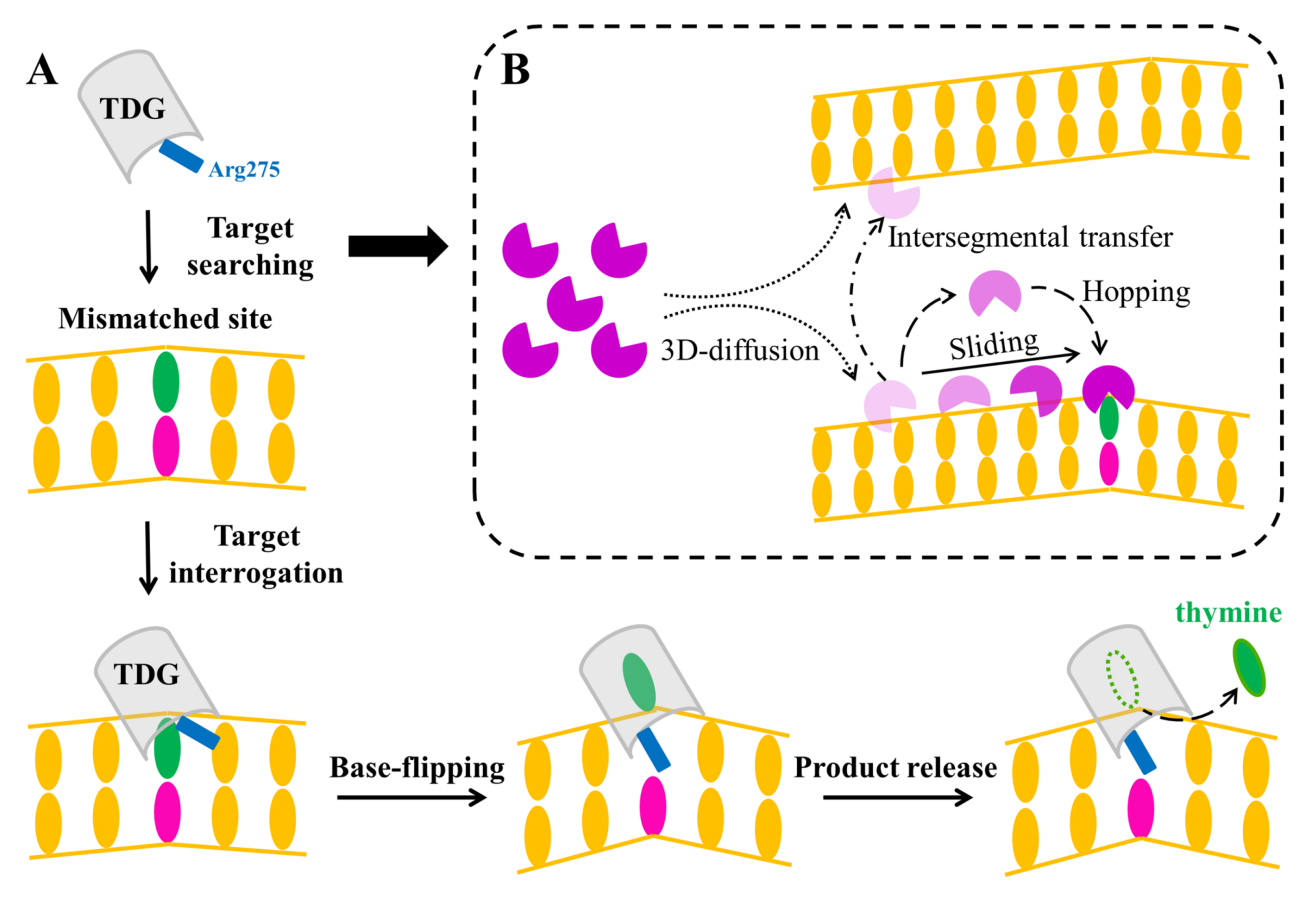
Figure 1 Schematic diagram of target searching and recognition mechanism underlying the base excision repair process performed by TDG (A) Sequential conformational changes are required in the TDG-involved base excision repair process, including target-site searching/interrogation, base-flipping of the target nucleobase, and product release following catalysis. Notably, Arg275, the intercalation residue in TDG (illustrated as a blue rectangular), was found to stabilize the partially flipped nucleobase at the early stage of base eversion, which further promotes the subsequent base-flipping process. Moreover, Arg275 could occupy the void space left by the completely flipped nucleobase, thereby locks the TDG-DNA complex in a fully base-flipped state. (B) A facilitated diffusion mechanism has been proposed to describe the target-searching process performed by TDG, whereby a combination of 3D-diffusion, intersegmental transfer, and short-range sliding/hopping along the DNA strand might take place.

The base excision by TDG can be broadly viewed as a multi-step process, namely target searching, base-flipping, cleavage of the N-glycosidic bond and product release ( Figure 1A). As a repair enzyme, TDG is required to firstly pinpoint the target nucleobases among millions of canonical ones in a highly efficient way. Considering genome folding and crowding effects imposed by the cellular environment, a combination of 3D random collision, intersegmental transfers, sliding, and hopping, has been proposed as a highly efficient target-searching mechanism ( Figure 1B) [15]. After locating the target sites, TDG adopts a base-flipping mechanism to finally recognize the nucleobases in the active site, as also observed for many DNA-binding enzymes, including methyltransferases ( *e*. *g*., M. *Hha*I, M. *Hae*III, and M. *Taq*I), other DNA glycosylases [ *e*. *g*., human alkyladenine DNA glycosylase (AAG), bacterial MutM, human OGG1, human uracil DNA glycosylase (UDG), and T4 EndoV], and endonucleases ( *e*. *g*., *E*. *coil* endonuclease IV and human APE1) [ 16– 27] . Then, the catalysis takes place by employing one water molecule as a nucleophile to attack the anomeric carbon C1′, resulting in the cleavage of the N-glycosidic bond between the target base and sugar [4]. Finally, the excised nucleobase is released from the TDG active-site, and the resulting TDG-DNA complex is then handed over to other BER enzymes.

Former experimental studies have successively resolved structures of several static TDG-DNA complexes involved in the abovementioned base excision process. The first glimpse of the TDG-bound DNA complex was obtained by Maiti’s group in 2008 [28]. In this structure, TDG is trapped to bind with either specific or non-specific DNA chain, and the latter likely represents a form that TDG interrogates the nucleobases before base flipping. While in the specific complex, TDG binds to one DNA duplex with a flipped abasic nucleotide (AP-site), and one critical intercalation residue Arg275 can penetrate into the base step and occupy the space vacated by the flipped nucleobase. This structure thus corresponds to the product complex right after the catalysis. Notably, the follow-up studies have successively trapped the base-flipped TDG-DNA complexes for various substrates (or analogues), including the G:U or G:T mismatch, 5caC, 5hmU, 5fC, U ^F^, β-F-5caC, and dfC ^F^ [ 11, 29– 35] . These static TDG-DNA structures provide insights into the critical structural elements in TDG responsible for the substrate binding and catalysis.

**Figure 2:**
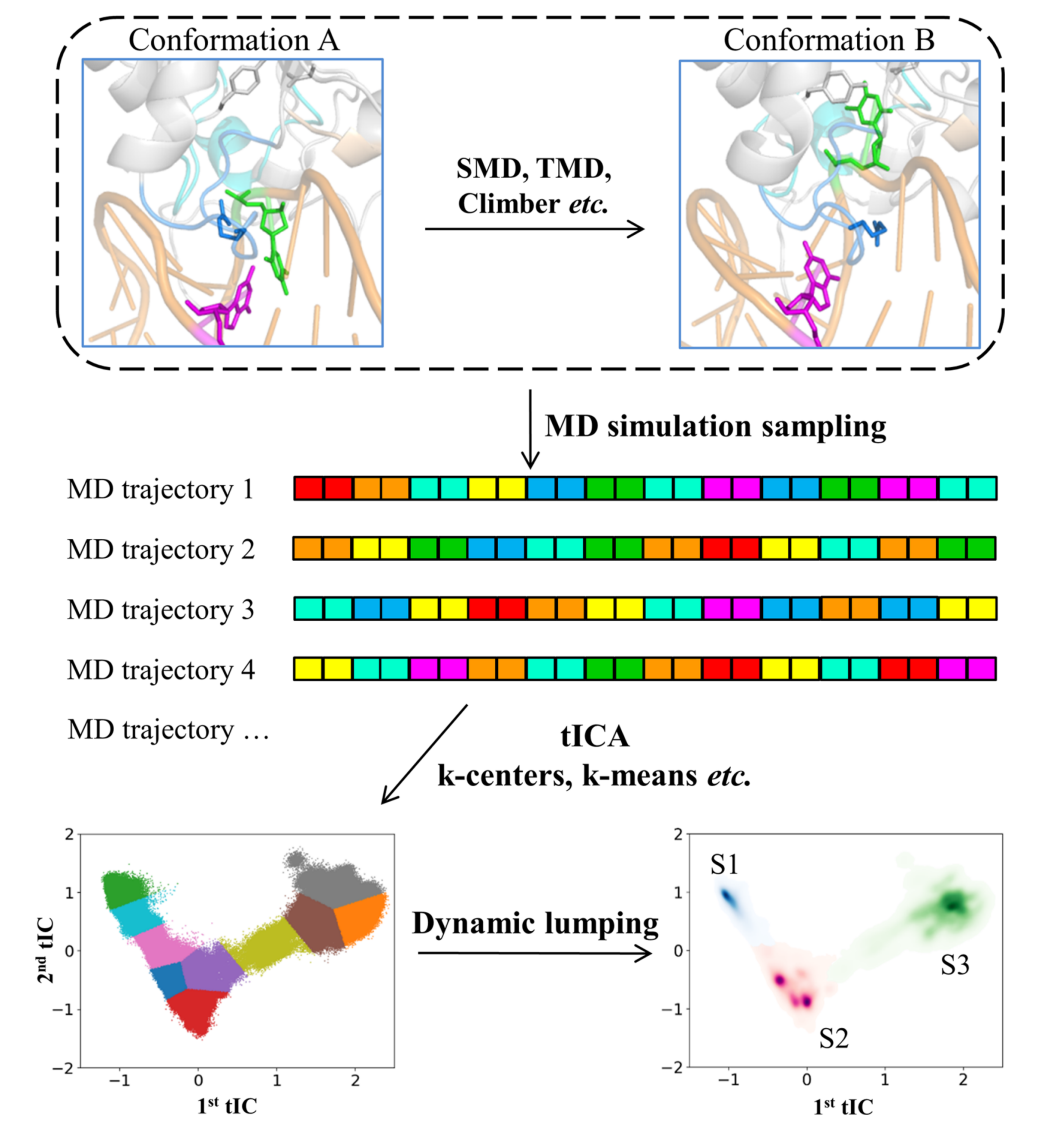
Figure 2 One general flowchart for MSM construction To investigate certain structural changes, one can firstly generate an initial conformational change pathway using biased simulation methods, such as steered molecular dynamics (SMD), targeted molecular dynamics (TMD), and Climber. Then, extensive unbiased molecular dynamics (MD) simulations can be performed to explore the phase space along the above initial transition path. The collected simulation dataset can be finally used to construct MSM, that is, clustering the MD conformations into hundreds of microstates using time-structure independent components analysis (tICA) and k-centers/k-means, etc., followed by lumping the microstates into several macrostates according to their kinetic properties.

Despite the previous efforts on elucidating the structural features of TDG, the conformational dynamics of TDG involved in various stages of DNA repair process is still inaccessible using experimental techniques under limited spatial-temporal resolution. Molecular dynamics (MD) simulation, as a powerful computational tool, has been applied to investigate critical conformational dynamics of extensive biomolecular systems at atomic resolution. Notably, one can now explore comparatively long-timescale dynamics *i*. *e*., hundreds of microseconds of certain structural changes for complex biomolecules by constructing Markov state model (MSM) [ 36– 40] . The general pipline for MSM construction is shown in Figure 2. MSM decomposes conformational space sampled from MD simulations into a set of microstates, where transitions within each state are relatively fast comparing to the inter-state transitions [ 41– 46] . This separation of timescales allows the construction of a Markovian model, in which the probability of transiting from state i to state j depends only on the identity of i but not previously visited states. MSM can be built from extensive short MD trajectories ( *e*. *g*., hundreds of nanoseconds MD simulations), and dynamics obtained from these short MD simulations can then be propagated to a longer timescale based on the following equation:

**Table:** 

$$\begin{array}{lr} P(n\Delta t)={\left[T(\Delta t)\right]}^{n}P(0) & \end{array}$$

where *P(n∆t)* is a vector of state populations at time n∆t and T is the transition probability matrix. In recent years, MSM has been successfully applied to elucidate conformational mechanisms of many biological molecules [ 47– 59] .

In this review, we summarize current understandings and our recent studies of the conformational dynamics of TDG involved in the DNA repair process from the perspective of computational simulations [ 60– 62] . The focused subjects include how TDG searches for its targets when approaching the lesions, how DNA conformation, *i*. *e*., DNA bending, minor-groove width, and roll angle, affects the TDG recognition, and what key structural motifs in TDG are responsible for recognizing certain DNA deformation. We will also discuss molecular mechanisms of detailed conformational dynamics of the base-flipping and excised-thymine release processes. More in-depth reviews regarding the catalysis and biological functions of TDG can be found elsewhere [ 6, 63] .

## Target-searching Mechanism of TDG

How the DNA-binding protein searches for its target site among millions of normal nucleobases is an intriguing research focus for extensive experimental and theoretical studies. A widely accepted model called “facilitated diffusion” has been proposed to describe the dynamics of target-searching proteins [ 15, 64– 69] . That is, proteins first bind to nonspecific site on DNA via 3D diffusion, likely impacted by crowded cellular environments. Then, sliding or hopping motions of protein along DNA takes place until the protein finally locates the target-site. The sliding involves constant association between protein and DNA, while the hopping requires transient micro-dissociation of DNA-binding protein from DNA and rebinding at a few base pairs (bps) apart. In addition, intersegmental transfers can also be a possible strategy whereby protein can transfer to other DNA strands or the same DNA molecule across a large bp interval.

To date, many experimental techniques, such as NMR [70], biochemical [ 71– 74] and single-molecular fluoresces studies [ 75, 76] , have been utilized to investigate the target-searching mechanisms of DNA glycosylases. Early biochemical work indicated that the UDG employs a processive mode for lesion search [77]. Likewise, AAG can also undergo a 1D-diffusion motion along DNA, and increasing the ion concentration can profoundly impact on the efficiency of search [78]. Intriguingly, AAG can bypass the road-blocker molecule *Eco*RI and bind to other target sites [79]. Similar results have also been observed for other DNA glycosylases, including methyl-CpG-binding domain protein 4 (MBD4) [80], adenine DNA glycosylase (MutY) and bacterial formamidopyrimidine-DNA glycosylase (Fpg or MutM) [81]. In particular, Stivers’s group designed a “molecular clock” strategy to differentiate the associative/dissociative diffusion and applied this method to UDG and hOGG1 [ 71, 73] . Their results suggest that both systems can slide along DNA via 1D translocation mode within a few bp range, although this finding is different from the former single-molecule fluorescence study [73]. Moreover, further *in vivo* assays revealed that the cellular environments, such as the ion concentration and microenvironments, can significantly influence the diffusion rate of protein along DNA [82]. In addition, the presence of nucleosomes can also largely affect the DNA-repair efficiency [ 83– 85] .

Considering the similar structural folds between TDG and UDG, it can be expected that TDG might be also capable of sliding along DNA in an associative mode, as previously observed for UDG [71]. Recent atomic force microscopy (AFM) and fluorescence studies showed that the TDG binding can bend both non-specific and specific DNA chain at two dominant conformations, with a bend angle of ~30° and ~60°, respectively [86]. Specifically, the former conformation is also present for the lesion-containing DNA before TDG binding, suggesting that the 30°-conformation is an intrinsic property of the free DNA, and the TDG binding can further induce DNA to a more severely bended conformation. Despite the extensive structural studies of TDG in complex with various mismatched/damaged nucleobases [ 29– 35] , the detailed mechanisms of how TDG scans and targets to the lesion sites before base-flipping remain elusive. Previous studies have obtained one TDG-DNA complex where the interrogating bp is an intrahelical form and also a canonical bp (PDB id: 2rba). Therefore, the 2rba structure is unable to reveal the true binding process between TDG and DNA prior to the base extrusion [28].

**Figure 3:**
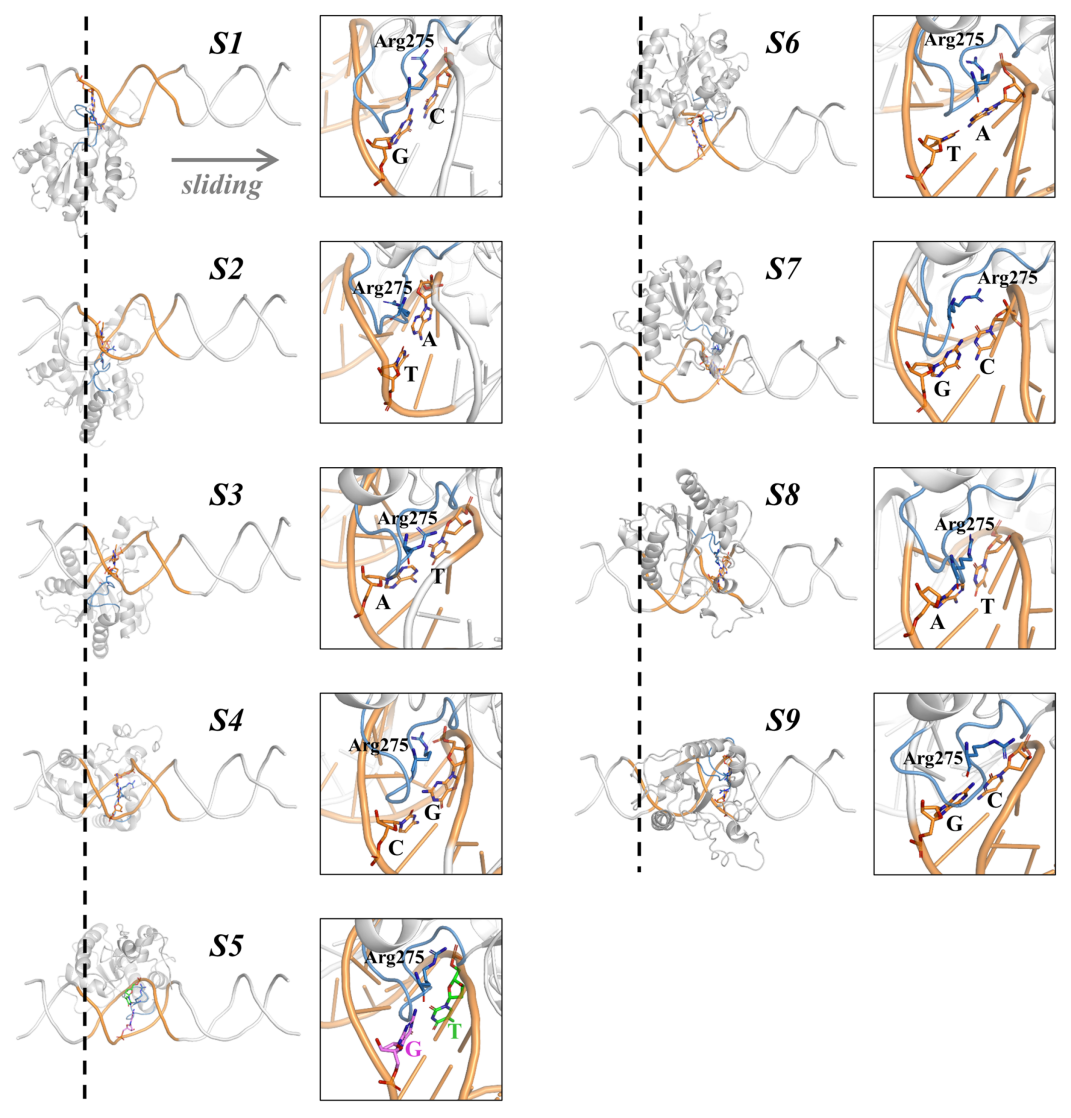
Figure 3 Nine metastable states (S1–S9) identified by MSM during the target-searching process performed by TDG Representative conformations for S1–S9 are shown. The representative conformation of each metastable state was randomly selected from the most populated microstates belonging to this state. For each state, the investigated 9-base pairs (bp) are shown in orange cartoon and the interrogated bp is depicted in orange sticks except that the mismatched G:T in S5 is highlighted with violet and green sticks. The intercalation loop of TDG is highlighted with blue cartoon. The zoomed-in view of intercalation loop is shown on the right panel, with the key intercalated residue Arg275 highlighted with blue sticks. In addition, the sliding direction of TDG and the starting point (at S1) are labeled by a grey arrow and black dashed line, respectively. The figures are modified from ref. 60.

To reveal how TDG locates to the target-site when approaching the lesions, we investigated a rotation-coupled sliding dynamic process of TDG along a 9-bp DNA segment that contains a G:T mismatch by constructing MSMs based on extensive unbiased MD simulations [60] ( Figure 3). We firstly built nine TDG-bound DNA complexes wherein TDG binds at varied bp-site. We then performed a number of targeted MD (TMD) simulations to derive an initial TDG sliding pathway along DNA. The resulting TMD trajectories were subject to extensive unbiased MD simulations. Around 25 microsecond MD simulations dataset was finally collected and MSM was constructed. The MSM results clearly identify nine metastable states, and for each state TDG locates at a certain bp-site. Thus, the transition of TDG between two adjacent bp sites is expected to overcome an energy barrier. Notably, the thermodynamically most favorable state is the conformations where TDG targets to the G:T mispair. From a kinetics view, TDG is found to diffuse rapidly when it is distant from the target-site, whereas it slows down as it approaches the mispaired site. This perturbed sliding rate of TDG is originated from the profound structural changes of TDG induced by the altered interacting interfaces between TDG and DNA during the process of locating the target.

Particularly, one key intercalation loop in TDG (residues Ala274–Ala277) can switch between various conformations during the sliding process [60]. When TDG locates at the nonspecific sites, the intercalation loop tends to insert shallowly into the DNA minor-groove, thereby adopting more flexible and solvent-exposed conformations. However, as TDG gets close to the target-site, the intercalation loop penetrates deeply into the DNA minor-groove by exhibiting a conformation resembling to the crystal structures ( *e*. *g*., 2rba). This interrogating loop-conformation potentially promotes the opening of the G:T mispair, DNA bending and widening of the minor-groove. In addition, two nearby TDG residues, Phe252 and Tyr288, are found to stabilize the specific loop-conformation via nonpolar interactions. Moreover, three positively charged TDG residues, *i*. *e*., Lys161, Lys232, and Arg281, serve as key electrostatic anchor points for facilitating the TDG transfer between adjacent bp-sites. That is, TDG tends to establish stable interactions via the above three residues with the DNA backbones when interrogating a certain bp site, whereas transiting to the adjacent sites necessitate the break of these salt-bridge contacts, resulting in loosed interactions between TDG and DNA. Our computational modeling therefore warrants further experimental tests.

It is noteworthy that the observed conformational switches of TDG during target searching have also been highlighted in other DNA-binding proteins, *e*. *g*., AAG [87], hOGG1 [ 23, 73, 75] , and UDG [ 88, 89] . In specific, a two-state model (open and closed) has been proposed to describe the interplays between protein and DNA, whereby the nonspecific binding favors an ‘open’ conformation of the DNA-targeting proteins. Meanwhile, binding to the specific site leads to a stable and ‘closed’ protein conformation [ 88, 89] . Additionally, the TDG binding at the target site can profoundly bend the DNA backbone at ~20°, which is consistent with former simulations result that TDG prefers to recognize a DNA conformation that bends at ~20° [90]. In this work, by constructing several TDG-DNA complexes with varied DNA bending angles (ranging from 0° to 60°), we discovered the key TDG residues responsible for recognizing certain bended DNA conformation. More strikingly, scrutinized structural analyses indicate that the roll-angle patterns of consecutive bps are well correlated with the DNA bend angle. This work therefore provides structural insights into the molecular mechanisms underlying the TDG-DNA recognition before base-flipping.

## Base-flipping Dynamics of TDG Substrates

When locating the lesions, TDG adopts a base-flipping strategy to extrude the target nucleobases from the DNA duplex into the TDG active-site, as observed in many DNA-binding proteins [ 21– 25] . The base eversion is accompanied by the deep insertion of the TDG intercalation-loop into the DNA minor-groove. In particular, the key loop-residue Arg275 can occupy the void space left by the flipped base, thereby preventing the flipped base swinging back to the DNA duplex. Early studies have demonstrated that the intrahelical nucleobases can spontaneously flip from DNA helix, even for the canonical bps [ 91– 93] . Introduction of the mismatched bp would significantly promote the base-flipping event [94]. It can be expected that the presence of DNA-binding proteins can impose significant structural distortions in DNA ( *e*. *g*., backbone bending and bp opening *etc*.), which in turn facilitates the base-extrusion process [ 29, 95, 96] . As described above, although many base-flipped TDG-DNA complexes have been obtained using crystallographic methods, the TDG-DNA complex before base-flipping and the complete base-flipping dynamics remain unclear.

**Figure 4:**
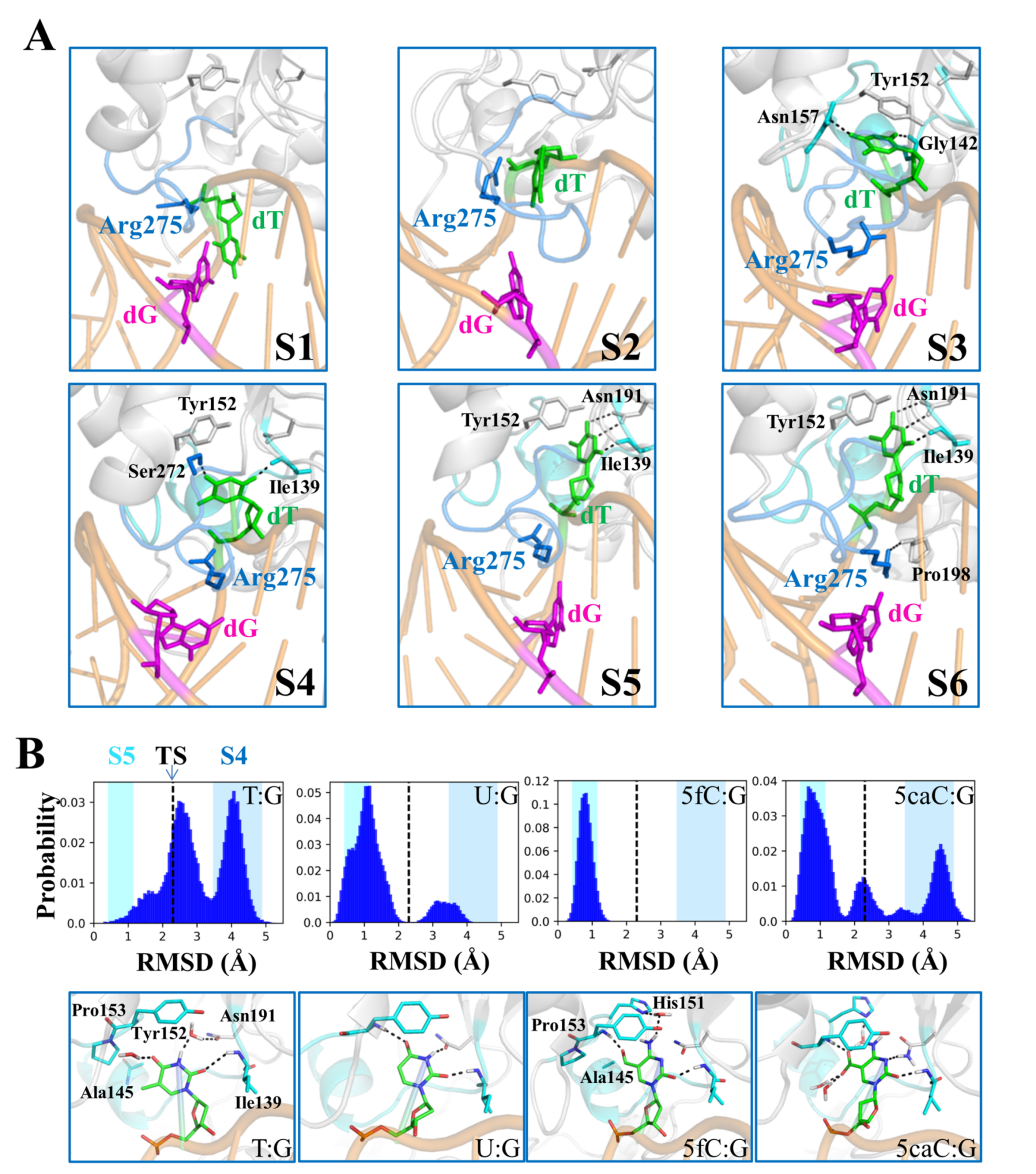
Figure 4 Base-flipping dynamics for TDG revealed by MSM (A) Representative conformations of six metastable states (S1-S6) in the base extrusion process. The representative conformation of each metastable state was randomly selected from the most populated microstates belonging to this state. The key residues that interact with the mismatched dT are shown in sticks. The hydrogen bonds are highlighted with dashed lines. (B) Histograms of the RMSD of the flipped base and representative conformation. The RMSD is calculated relative to the minimized recognition complex for each TDG substrate, including dT, dU, 5fC, and 5caC. The RMSD of conformations from transition state (TS) is labeled with black dashed line in each plot. In the background, the fluctuation of the RMSD values calculated based on the MSM is represented by colored boxes for S4 in blue and S5 in cyan. The conformation near to the TS is shown for dT, whereas the flipped states are chosen for other three substrates. Figures are modified from ref. 61.

One interrogated complex of TDG-DNA structure where TDG inspects an intrahelical bp was built by computational modeling and employed as the starting structure to generate initial base-flipping pathways [61]. A series of MSMs were then constructed to reveal the complete base-flipping dynamics of one mismatched thymine exerted by TDG based on extensive MD simulations. The resulting MSM captured the key intermediates of the flipped thymine during the complete base-extrusion process, and revealed the critical TDG residues responsible for mediating the inter-state transitions, including the residues Gly142, Asn157, Ser272, Ser273, and Cys276 ( Figure 4A). Additional comparison studies were conducted to evaluate how different TDG substrates, i.e., dU, 5fC, and 5caC, influence the base-flipping dynamics. The transition-state analyses suggest that the base-flipping rates for various nucleobases likely follow an order of dU≈5fC>5caC>dT, owning to the varied chemical groups ( Figure 4B). The above findings not only comply with existing experimental evidence [ 29, 97] , but also potentiate additional experimental validation.

One intriguing question for the base-flipping study is whether the proteins recognize the target nucleobases in an active or a passive mode. The active mode describes a searching mechanism whereby the protein firstly inspects an intrahelical bp, then proactively promotes the base-flipping. An alternative scenario is that the spontaneously flipped nucleobases can be transiently captured by the searching proteins (passive mode). Former NMR work has suggested a passive searching mechanism for UDG [70]. An active base-extrusion, however, has been proposed for Fpg/MutM using single-molecular and crystallographic techniques, and also for bacterial 3-methyladenine glycosylase (AlkA) and MutY [ 98, 99] . Likewise, our computational work supports an active involvement of TDG in triggering the base flipping. Specifically, the intercalated residue Arg275 plays an essential role in stabilizing the partially flipped thymine via cation-π interactions, resulting in a low energetic flipping path along the DNA minor groove [61].

## Dynamics of the Product Release from TDG Active-site after Catalysis

Upon entering into the TDG active-site, the flipped nucleobase can be cleaved by employing one water molecule as nucleophile [ 1, 100, 101] . Then, the excised base has to be released from the active site to generate a stable TDG-DNA complex with an AP-site. The bound TDG is responsible for protecting the AP-site from undesired damages and recruiting other BER enzymes. Former experimental efforts have endeavored to trap the tertiary TDG complex after catalysis, however, all failed to capture any excised base in the active site, suggesting that the product is prone to dissociating from the binding pocket right after the cleavage [ 31, 33, 34] . Nevertheless, potential product-releasing channels have been proposed by former crystallographic studies [31].

**Figure 5:**
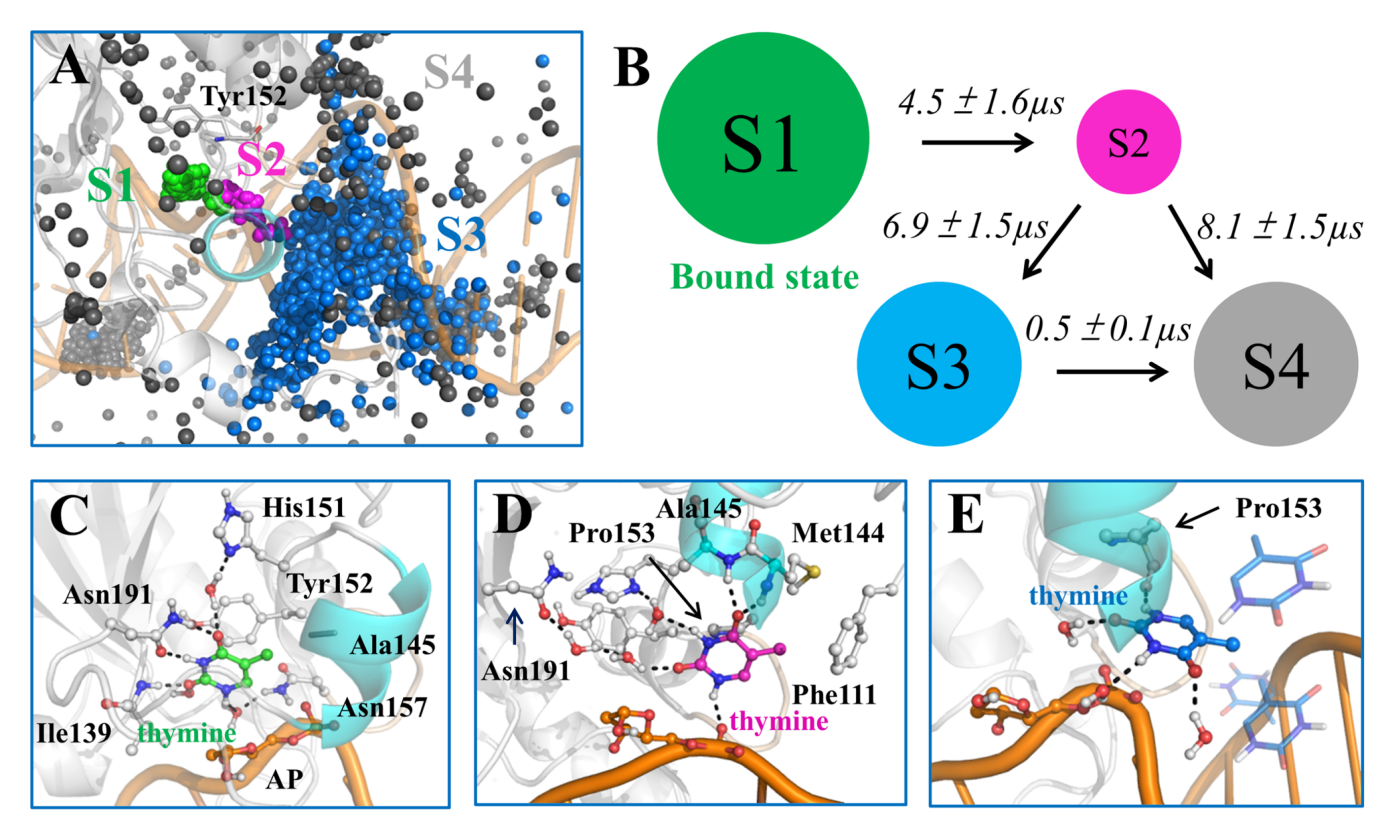
Figure 5 Product release dynamics from TDG active site revealed by MSM (A) For each state, the conformations of the product were randomly chosen according to their stationary distributions. For each thymine conformation, only C2 atom is shown in sphere. The active-site residue Tyr152 is also shown as a reference point. (B) The 4-states kinetic network derived from the MSM. The mean first passage time (MFPT) for the forward transition is labeled above each arrow. (C–E) Selected representative conformation for S1 (C), S2 (D), and S3 (E). The structure was randomly selected from the largest microstate for each macrostate. Key residues and water molecules that interact with thymine are shown in sticks, and the hydrogen bonds between thymine and neighboring molecules are highlighted with dashed lines. The figures are derived from ref. 62.

Computational simulations have been conducted to identify the key intermediates of the excised thymine as well as to demonstrate complete product-release pathway and the associated thermodynamic/kinetic properties [62]. The constructed MSM revealed the detailed interaction networks between the product and TDG-DNA complex, and pinpointed the rate-limiting transition during the whole product release process ( Figure 5A,B). Moreover, structural inspections demonstrated strong interplays between TDG and DNA chains that, via a conformational selection mode, facilitate the transfer of excised thymine through the narrow releasing channel ( Figure 5C–E) [62]. More intriguingly, the study identified a key TDG residue, Gly142, as a gating residue lying along the product release pathway. Gly142 therefore serves as a potential substitution site to trap the product in the active site. In our previous study, we performed free energy calculations to evaluate the relative product-releasing rates between the wild-type (WT) and the G142Y mutant [62]. Specifically, the potential of mean force along the dominant releasing path was calculated by conducting steered molecular dynamics simulation with constant velocity for each system. The distance between the center of mass of the product and one backbone P atom of DNA was defined as the reaction coordinate (RC). The final free energy profiles were obtained based on the Jarzynski’s equality that evaluates the free energy difference between two states from the performed work [102]. The results indicate that the G142Y mutant shows a higher transition barrier during the product release process than the WT, with a free-energy difference of ~7.3 *k _B_* T, which corresponds to ~1000-fold decrease in the transition rate. This work thus provides a potential solution to successively obtain the tertiary complex using experimental techniques.

To examine how different bases ( *e*. *g*., U, 5hmU, 5fC, and 5caC) impact the release kinetics, the ideal way is to construct MSM for each base system as we did for the G:T mispair, which, however, requires tremendous computational resources to construct a reliable kinetic model. Instead, Da *et al*. [62] evaluated the relative product-releasing rates for the above TDG substrates, by employing the thymine as a reference system. For each given system, the RC and detailed setups for free-energy calculation are the same as those used for the G142Y mutant. The results show that U, 5caC, and 5hmU exhibit relatively faster product releasing rate than thymine, whereas 5fC displays similar release kinetics. Such a methodology thus provides an efficient way to evaluate the relative releasing rates for various TDG substrates. Taken together, the computational work demonstrates that the overall releasing time for thymine is ~10 μs. Considering the similar structural fold of TDG to that of UDG, we hypothesize that product-releasing mechanism identified in TDG is likely universal among different UNG members.

## Conclusion Remarks

Here, we reviewed the current progress towards understanding the critical conformational dynamics of TDG involved in the BER pathway in a perspective of computational simulations. The main focus includes the searching dynamics of TDG along DNA that contains one specific target-site, base-flipping dynamics of one thymine nucleotide promoted by TDG, and the product release process after the catalysis with TDG. By constructing the kinetic models, we are able to identify the key intermediate states involved in the conformational transitions and pinpoint the critical structural elements and residues responsible for regulating the state-to-state transitions. As a result, the theoretical and computational work support designing new experimental tests for further model validations. Despite that, there are some limitations of the current computational studies. In the target-searching work, only one possible searching mechanism, *i*. *e*., rotation-coupled sliding motion, is investigated. Alternative scenario, such as the hopping model, has not been taken into account. Therefore, comparisons of different searching strategies are yet to be achieved. In addition, we examined the relative kinetics of various TDG substrates for the base-flipping or product-release process, by assuming that all the substrates undergo similar transition paths. The assumption, however, is not necessarily true, as different TDG substrates with varied chemical groups may exhibit totally different conformational transitions. Additional efforts are needed to develop cutting-edge computation methods to explore protein-DNA conformational space in a more efficient way, for example, using machine learning method, etc.

## References

[1] Schormann N, Ricciardi R, Chattopadhyay D (2014). Uracil-DNA glycosylases-Structural and functional perspectives on an essential family of DNA repair enzymes. Protein Sci.

[2] Tomasetti C, Li L, Vogelstein B (2017). Stem cell divisions, somatic mutations, cancer etiology, and cancer prevention. Science.

[3] Dianov GL, Hübscher U (2013). Mammalian base excision repair: the forgotten archangel. Nucleic Acids Res.

[4] Schermerhorn KM, Delaney S (2014). A chemical and kinetic perspective on base excision repair of DNA. Acc Chem Res.

[5] Stivers JT, Jiang YL (2003). A mechanistic perspective on the chemistry of DNA repair glycosylases. Chem Rev.

[6] Drohat AC, Maiti A (2014). Mechanisms for enzymatic cleavage of the N-glycosidic bond in DNA. Org Biomol Chem.

[7] Bennett MT, Rodgers MT, Hebert AS, Ruslander LE, Eisele L, Drohat AC (2006). Specificity of human thymine DNA glycosylase depends on
*n*-glycosidic bond stability. J Am Chem Soc.

[8] Liu P, Burdzy A, Sowers LC (2003). Repair of the mutagenic DNA oxidation product, 5-formyluracil. DNA Repair.

[9] Morgan MT, Bennett MT, Drohat AC (2007). Excision of 5-halogenated uracils by human thymine DNA glycosylase. J Biol Chem.

[10] He YF, Li BZ, Li Z, Liu P, Wang Y, Tang Q, Ding J (2011). Tet-mediated formation of 5-carboxylcytosine and its excision by TDG in mammalian DNA. Science.

[11] Pidugu LS, Dai Q, Malik SS, Pozharski E, Drohat AC (2019). Excision of 5-carboxylcytosine by thymine DNA glycosylase. J Am Chem Soc.

[12] Mancuso P, Tricarico R, Bhattacharjee V, Cosentino L, Kadariya Y, Jelinek J, Nicolas E (2019). Thymine DNA glycosylase as a novel target for melanoma. Oncogene.

[13] Kolendowski B, Hassan H, Krstic M, Isovic M, Thillainadesan G, Chambers AF, Tuck AB (2018). Genome-wide analysis reveals a role for TDG in estrogen receptor-mediated enhancer RNA transcription and 3-dimensional reorganization. EpiGenet Chromatin.

[14] Yan JB, Lai CC, Jhu JW, Gongol B, Marin TL, Lin SC, Chiu HY (2020). Insulin and metformin control cell proliferation by regulating tdg-mediated dna demethylation in liver and breast cancer cells. Mol Ther - Oncolytics.

[15] Esadze A, Stivers JT (2018). Facilitated diffusion mechanisms in DNA base excision repair and transcriptional activation. Chem Rev.

[16] Klimasauskas S, Kumar S, Roberts RJ, Cheng X (1994). Hhal methyltransferase flips its target base out of the DNA helix. Cell.

[17] O′Gara M, Horton JR, Roberts RJ, Cheng X (1998). Structures of HhaI methyltransferase complexed with substrates containing mismatches at the target base. Nat Struct Mol Biol.

[18] Reinisch KM, Chen L, Verdine GL, Lipscomb WN (1995). The crystal structure of Haelll methyltransferase covalently complexed to DNA: An extrahelical cytosine and rearranged base pairing. Cell.

[19] Goedecke K, Pignot M, Goody RS, Scheidig AJ, Weinhold E (2001). Structure of the N6-adenine DNA methyltransferase M.TaqI in complex with DNA and a cofactor analog. Nat Struct Biol.

[20] Blumenthal RM, Cheng X (2001). A Taq attack displaces bases. Nat Struct Biol.

[21] Lau AY, Schärer OD, Samson L, Verdine GL, Ellenberger T (1998). Crystal structure of a human alkylbase-DNA repair enzyme complexed to DNA. Cell.

[22] Fromme JC, Verdine GL (2003). DNA lesion recognition by the bacterial repair enzyme MutM. J Biol Chem.

[23] Bruner SD, Norman DPG, Verdine GL (2000). Structural basis for recognition and repair of the endogenous mutagen 8-oxoguanine in DNA. Nature.

[24] Slupphaug G, Mol CD, Kavli B, Arvai AS, Krokan HE, Tainer JA (1996). A nucleotide-flipping mechanism from the structure of human uracil–DNA glycosylase bound to DNA. Nature.

[25] Morikawa K, Matsumoto O, Tsujimoto M, Katayanagi K, Ariyoshi M, Doi T, Ikehara M (1992). X-ray structure of T4 endonuclease V: An excision repair enzyme specific for a pyrimidine dimer. Science.

[26] Hosfield DJ, Guan Y, Haas BJ, Cunningham RP, Tainer JA (1999). Structure of the DNA repair enzyme endonuclease IV and its DNA complex. Cell.

[27] Mol CD, Izumi T, Mitra S, Tainer JA (2000). DNA-bound structures and mutants reveal abasic DNA binding by APE1 DNA repair and coordination. Nature.

[28] Maiti A, Morgan MT, Pozharski E, Drohat AC (2008). Crystal structure of human thymine DNA glycosylase bound to DNA elucidates sequence-specific mismatch recognition. Proc Natl Acad Sci U S A.

[29] Maiti A, Noon MS, MacKerell Jr. AD, Pozharski E, Drohat AC (2012). Lesion processing by a repair enzyme is severely curtailed by residues needed to prevent aberrant activity on undamaged DNA. Proc Natl Acad Sci U S A.

[30] Coey CT, Malik SS, Pidugu LS, Varney KM, Pozharski E, Drohat AC (2016). Structural basis of damage recognition by thymine DNA glycosylase: Key roles for N-terminal residues. Nucleic Acids Res.

[31] Malik SS, Coey CT, Varney KM, Pozharski E, Drohat AC (2015). Thymine DNA glycosylase exhibits negligible affinity for nucleobases that it removes from DNA. Nucleic Acids Res.

[32] Zhang L, Lu X, Lu J, Liang H, Dai Q, Xu GL, Luo C (2012). Thymine DNA glycosylase specifically recognizes 5-carboxylcytosine-modified DNA. Nat Chem Biol.

[33] Hashimoto H, Zhang X, Cheng X (2013). Activity and crystal structure of human thymine DNA glycosylase mutant N140A with 5-carboxylcytosine DNA at low pH. DNA Repair.

[34] Hashimoto H, Hong S, Bhagwat AS, Zhang X, Cheng X (2012). Excision of 5-hydroxymethyluracil and 5-carboxylcytosine by the thymine DNA glycosylase domain: its structural basis and implications for active DNA demethylation. Nucleic Acids Res.

[35] Pidugu LS, Flowers JW, Coey CT, Pozharski E, Greenberg MM, Drohat AC (2016). Structural basis for excision of 5-formylcytosine by thymine DNA glycosylase. Biochemistry.

[36] Da LT, Wang D, Huang X (2012). Dynamics of pyrophosphate ion release and its coupled trigger loop motion from closed to open state in RNA polymerase II. J Am Chem Soc.

[37] Unarta IC, Zhu L, Tse CKM, Cheung PPH, Yu J, Huang X (2018). Molecular mechanisms of RNA polymerase II transcription elongation elucidated by kinetic network models. Curr Opin Struct Biol.

[38] Da LT, Pardo Avila F, Wang D, Huang X (2013). A two-state model for the dynamics of the pyrophosphate ion release in bacterial RNA polymerase. PLoS Comput Biol.

[39] Silva DA, Weiss DR, Pardo Avila F, Da LT, Levitt M, Wang D, Huang X (2014). Millisecond dynamics of RNA polymerase II translocation at atomic resolution. Proc Natl Acad Sci U S A.

[40] Da LT, Pardo-Avila F, Xu L, Silva DA, Zhang L, Gao X, Wang D (2016). Bridge helix bending promotes RNA polymerase II backtracking through a critical and conserved threonine residue. Nat Commun.

[41] Bowman GR, Huang X, Pande VS (2009). Using generalized ensemble simulations and Markov state models to identify conformational states. Methods.

[42] Shukla D, Hernández CX, Weber JK, Pande VS (2015). Markov state models provide insights into dynamic modulation of protein function. Acc Chem Res.

[43] Zhu L, Sheong FK, Zeng X, Huang X (2016). Elucidation of the conformational dynamics of multi-body systems by construction of Markov state models. Phys Chem Chem Phys.

[44] Chodera JD, Noé F (2014). Markov state models of biomolecular conformational dynamics. Curr Opin Struct Biol.

[45] Prinz JH, Wu H, Sarich M, Keller B, Senne M, Held M, Chodera JD (2011). Markov models of molecular kinetics: generation and validation. J Chem Phys.

[46] Noé F, Fischer S (2008). Transition networks for modeling the kinetics of conformational change in macromolecules. Curr Opin Struct Biol.

[47] Zhuang W, Cui RZ, Silva DA, Huang X (2011). Simulating the T-jump-triggered unfolding dynamics of trpzip2 peptide and its time-resolved IR and two-dimensional IR signals using the markov state model approach. J Phys Chem B.

[48] Voelz VA, Bowman GR, Beauchamp K, Pande VS (2010). Molecular simulation of
*ab Initio* protein folding for a millisecond folder NTL9(1−39). J Am Chem Soc.

[49] Bowman GR, Voelz VA, Pande VS (2011). Atomistic folding simulations of the five-helix bundle protein λ
_6−85_. J Am Chem Soc.

[50] Noé F, Schütte C, Vanden-Eijnden E, Reich L, Weikl TR (2009). Constructing the equilibrium ensemble of folding pathways from short off-equilibrium simulations. Proc Natl Acad Sci U S A.

[51] Kohlhoff KJ, Shukla D, Lawrenz M, Bowman GR, Konerding DE, Belov D, Altman RB (2014). Cloud-based simulations on Google Exacycle reveal ligand modulation of GPCR activation pathways. Nat Chem.

[52] Qiao Q, Bowman GR, Huang X (2013). Dynamics of an intrinsically disordered protein reveal metastable conformations that potentially seed aggregation. J Am Chem Soc.

[53] Bowman GR, Geissler PL (2012). Equilibrium fluctuations of a single folded protein reveal a multitude of potential cryptic allosteric sites. Proc Natl Acad Sci U S A.

[54] Lane TJ, Bowman GR, Beauchamp K, Voelz VA, Pande VS (2011). Markov state model reveals folding and functional dynamics in ultra-long MD trajectories. J Am Chem Soc.

[55] Huang X, Bowman GR, Bacallado S, Pande VS (2009). Rapid equilibrium sampling initiated from nonequilibrium data. Proc Natl Acad Sci U S A.

[56] Silva DA, Bowman GR, Sosa-Peinado A, Huang X (2011). A Role for both conformational selection and induced fit in ligand binding by the LAO protein. PLoS Comput Biol.

[57] Shukla D, Meng Y, Roux B, Pande VS (2014). Activation pathway of Src kinase reveals intermediate states as targets for drug design. Nat Commun.

[58] Choudhary OP, Paz A, Adelman JL, Colletier JP, Abramson J, Grabe M (2014). Structure-guided simulations illuminate the mechanism of ATP transport through VDAC1. Nat Struct Mol Biol.

[59] Kasson PM, Kelley NW, Singhal N, Vrljic M, Brunger AT, Pande VS (2006). Ensemble molecular dynamics yields submillisecond kinetics and intermediates of membrane fusion. Proc Natl Acad Sci U S A.

[60] Tian J, Wang L, Da LT (2021). Atomic resolution of short-range sliding dynamics of thymine DNA glycosylase along DNA minor-groove for lesion recognition. Nucleic Acids Res.

[61] Da LT, Yu J (2018). Base-flipping dynamics from an intrahelical to an extrahelical state exerted by thymine DNA glycosylase during DNA repair process. Nucleic Acids Res.

[62] Da LT, Shi Y, Ning G, Yu J (2018). Dynamics of the excised base release in thymine DNA glycosylase during DNA repair process. Nucleic Acids Res.

[63] Drohat AC, Coey CT (2016). Role of base excision “repair” enzymes in erasing epigenetic marks from DNA. Chem Rev.

[64] Halford SE, Marko JF (2004). How do site-specific DNA-binding proteins find their targets?. Nucleic Acids Res.

[65] Lomholt MA, van den Broek B, Kalisch SMJ, Wuite GJL, Metzler R (2009). Facilitated diffusion with DNA coiling. Proc Natl Acad Sci U S A.

[66] Hammar P, Leroy P, Mahmutovic A, Marklund EG, Berg OG, Elf J (2012). The
*lac* Repressor Displays Facilitated Diffusion in Living Cells. Science.

[67] Suter DM (2020). Transcription factors and DNA play hide and seek. Trends Cell Biol.

[68] Gowers DM, Wilson GG, Halford SE (2005). Measurement of the contributions of 1D and 3D pathways to the translocation of a protein along DNA. Proc Natl Acad Sci U S A.

[69] Sokolov IM, Metzler R, Pant K, Williams MC (2005). Target search of N sliding proteins on a DNA. Biophys J.

[70] Cao C, Jiang YL, Stivers JT, Song F (2004). Dynamic opening of DNA during the enzymatic search for a damaged base. Nat Struct Mol Biol.

[71] Schonhoft JD, Stivers JT (2012). Timing facilitated site transfer of an enzyme on DNA. Nat Chem Biol.

[72] Porecha RH, Stivers JT (2008). Uracil DNA glycosylase uses DNA hopping and short-range sliding to trap extrahelical uracils. Proc Natl Acad Sci U S A.

[73] Rowland MM, Schonhoft JD, McKibbin PL, David SS, Stivers JT (2014). Microscopic mechanism of DNA damage searching by hOGG1. Nucleic Acids Res.

[74] Hedglin M, Zhang Y, O′Brien PJ (2015). Probing the DNA structural requirements for facilitated diffusion. Biochemistry.

[75] Blainey PC, Luo G, Kou SC, Mangel WF, Verdine GL, Bagchi B, Xie XS (2009). Nonspecifically bound proteins spin while diffusing along DNA. Nat Struct Mol Biol.

[76] Blainey PC, van Oijen AM, Banerjee A, Verdine GL, Xie XS (2006). A base-excision DNA-repair protein finds intrahelical lesion bases by fast sliding in contact with DNA. Proc Natl Acad Sci U S A.

[77] Bennett SE, Sanderson RJ, Mosbaugh DW (1995). Processivity of
*Escherichia coli* and rat liver mitochondrial uracil-DNA glycosylase is affected by NaCl concentration. Biochemistry.

[78] Hedglin M, O′Brien PJ (2008). Human alkyladenine DNA glycosylase employs a processive search for DNA damage. Biochemistry.

[79] Hedglin M, O′Brien PJ (2010). Hopping enables a DNA repair glycosylase to search both strands and bypass a bound protein. ACS Chem Biol.

[80] Walavalkar NM, Cramer JM, Buchwald WA, Scarsdale JN, Williams Jr DC (2014). Solution structure and intramolecular exchange of methyl-cytosine binding domain protein 4 (MBD4) on DNA suggests a mechanism to scan for mCpG/TpG mismatches. Nucleic Acids Res.

[81] Francis AW, David SS (2003). *Escherichia coli* MutY and Fpg utilize a processive mechanism for target location. Biochemistry.

[82] Esadze A, Rodriguez G, Weiser BP, Cole PA, Stivers JT (2017). Measurement of nanoscale DNA translocation by uracil DNA glycosylase in human cells. Nucleic Acids Res.

[83] Hinz JM, Rodriguez Y, Smerdon MJ (2010). Rotational dynamics of DNA on the nucleosome surface markedly impact accessibility to a DNA repair enzyme. Proc Natl Acad Sci U S A.

[84] Zhang Y, O′Brien PJ (2015). Repair of alkylation damage in eukaryotic chromatin depends on searching ability of alkyladenine DNA glycosylase. ACS Chem Biol.

[85] Olmon ED, Delaney S (2017). Differential ability of five DNA glycosylases to recognize and repair damage on nucleosomal DNA. ACS Chem Biol.

[86] Buechner CN, Maiti A, Drohat AC, Tessmer I (2015). Lesion search and recognition by thymine DNA glycosylase revealed by single molecule imaging. Nucleic Acids Res.

[87] Setser JW, Lingaraju GM, Davis CA, Samson LD, Drennan CL (2012). Searching for DNA lesions: structural evidence for lower- and higher-affinity DNA binding conformations of human alkyladenine DNA glycosylase. Biochemistry.

[88] Schonhoft JD, Kosowicz JG, Stivers JT (2013). DNA translocation by human uracil DNA glycosylase: role of DNA phosphate charge. Biochemistry.

[89] Friedman JI, Majumdar A, Stivers JT (2009). Nontarget DNA binding shapes the dynamic landscape for enzymatic recognition of DNA damage. Nucleic Acids Res.

[90] Li S, Da LT (2020). Key structural motifs in thymine DNA glycosylase responsible for recognizing certain DNA bent conformation revealed by atomic simulations. Biochem Biophys Res Commun.

[91] Dallmann A, Dehmel L, Peters T, Mügge C, Griesinger C, Tuma J, Ernsting NP (2010). 2-Aminopurine incorporation perturbs the dynamics and structure of DNA. Angew Chem Int Ed.

[92] Folta-Stogniew E, Russu IM (1994). Sequence dependence of base-pair opening in a DNA dodecamer containing the CACA/GTGT sequence motif. Biochemistry.

[93] Leijon M, Gräslund A (1992). Effects of sequence and length on imino proton exchange and base pair opening kinetics in DNA oligonucleotide duplexes. Nucl Acids Res.

[94] Moe JG, Russu IM (1992). Kinetics and energetics of base-pair opening in 5′-d(CGCGAATTCGCG)-3′ and a substituted dodecamer containing G.cntdot.T mismatches. Biochemistry.

[95] Lyons DM, O′Brien PJ (2009). Efficient recognition of an unpaired lesion by a DNA repair glycosylase. J Am Chem Soc.

[96] Hendershot JM, O′Brien PJ (2014). Critical role of DNA intercalation in enzyme-catalyzed nucleotide flipping. Nucleic Acids Res.

[97] Maiti A, Michelson AZ, Armwood CJ, Lee JK, Drohat AC (2013). Divergent mechanisms for enzymatic excision of 5-formylcytosine and 5-carboxylcytosine from DNA. J Am Chem Soc.

[98] Bowman BR, Lee S, Wang S, Verdine GL (2010). Structure of
*Escherichia coli* AlkA in complex with undamaged DNA. J Biol Chem.

[99] Wang L, Chakravarthy S, Verdine GL (2017). Structural basis for the lesion-scanning mechanism of the MutY DNA glycosylase. J Biol Chem.

[100] Cortázar D, Kunz C, Saito Y, Steinacher R, Schär P (2007). The enigmatic thymine DNA glycosylase. DNA Repair.

[101] McCullough AK, Dodson ML, Lloyd RS (1999). Initiation of base excision repair: glycosylase mechanisms and structures. Annu Rev Biochem.

[102] Jarzynski C (1997). nonequilibrium equality for free energy differences. Phys Rev Lett.

